# Detection of prions in oocytes and ovaries of ewes naturally infected with classical scrapie

**DOI:** 10.1186/s13567-025-01512-0

**Published:** 2025-04-10

**Authors:** Paula A. Marco Lorente, Maialen Zinkunegi, Diego Sola, Nerea Larrañaga, Belén Marín, Bernardino Moreno, Juan J. Badiola, Rosa Bolea, Alicia Otero

**Affiliations:** Centro de Encefalopatías y Enfermedades Transmisibles Emergentes, Facultad de Veterinaria, Universidad de Zaragoza, Instituto Agroalimentario de Aragón-IA2, 50013 Saragossa, Spain

**Keywords:** Prion, PrP^Sc^, classical scrapie, vertical transmission, ovaries, oocytes

## Abstract

Classical scrapie is a prion disease that affects goats and sheep and is transmitted mainly horizontally. However, infection can also occur vertically, from mother to offspring, but the exact mechanism is unclear. The possibilities include transmission during pregnancy, birth, or after birth. Some studies have reported that prions are present in various reproductive tissues and fluids, suggesting that vertical transmission could occur through the germ line. However, the presence of prions in female reproductive tissues and oocytes has not been extensively studied, so their role in vertical transmission is unknown. Oocytes and ovarian tissues from 25 naturally infected ewes (ARQ/ARQ and VRQ/VRQ genotypes) and healthy controls were analysed. The samples were subjected to PMCA for prion detection. Histopathological and immunohistochemical evaluations of ovarian tissues were also performed. We demonstrated that PrP^Sc^ can accumulate in the oocytes and ovaries of scrapie-infected ewes, indicating a potential germline route of transmission. Prion accumulation appears to be influenced by the host genotype and prion strain, emphasizing the need for ultrasensitive detection techniques. Further research under controlled conditions is necessary to elucidate the mechanisms and implications for disease control and breeding programs.

**Supplementary Information:**

The online version contains supplementary material available at 10.1186/s13567-025-01512-0.

## Introduction

Scrapie is a transmissible spongiform encephalopathy (TSE) that naturally affects goats and sheep caused by the misfolding of the physiological protein PrP^C^ (cellular prion protein) into the pathological isoform PrP^Sc^, commonly known as prion, which is characterized by its self-replicating and infectious nature and its high resistance to radiation, heat, and proteases [1–3]. Owing to these properties, PrP^Sc^ cannot be degraded, leading to its accumulation and the formation of PrP^Sc^ deposits in various organs and tissues but primarily in the central nervous system (CNS), leading to a chronic neurodegenerative disorder with a slow progressive course and fatal outcome [4–6].

Classical scrapie is of particular concern to animal health because of its ability to spread within flocks. Transmission occurs mainly horizontally, either through direct contact between animals or indirectly through environmental contamination with prions released through excretions (faeces or urine), secretions (saliva or milk), carcasses, or placental remains of infected animals [7–10], the latter being considered the main source of contamination in this disease [11–13]. However, infection can also occur through vertical or maternal routes. There is still uncertainty as to the exact timing and route by which this occurs. Three possibilities are suggested: prenatal transmission (between oogenesis and delivery) [14–18], delivery (by contact with placenta or fluids) [7, 8, 13, 19] or after birth, which can occur by ingestion of contaminated colostrum or milk [20–23] or contact with the environment contaminated by the placenta and other fluids of the infected mother [12–14, 19, 24, 25].

To gain a clearer understanding of vertical transmission routes and their associated risk levels, several studies have been conducted on the accumulation of prions in various organs, tissues, fluids and cells associated with this route of transmission [9, 15, 18, 19, 26–29]. Thus, positive results have been reported in the uterus [16], placenta, placentomes [28, 30, 31], amniotic fluid, umbilical cord, foetuses [15, 16, 32], and even semen [9], suggesting the potential for vertical transmission through the germ line. However, to our knowledge, no study has been conducted on the presence of prions in oocytes from naturally scrapie-infected ewes, so it is unknown whether the female germline could play a role in the transmission of the disease. In addition, studies on the role of the ovary in scrapie are scarce, and the presence of PrP^Sc^ deposits in this organ has never been reported [26, 28, 33]. Nevertheless, studies on the ovaries of infected females from different species, such as Rocky Mountain elk, deer with chronic wasting disease (CWD) and even humans with variant Creutzfeldt-Jakob disease (vCJD), have yielded positive results [34–36], especially those that have resorted to ultrasensitive techniques such as protein misfolding cyclic amplification (PMCA) or real-time quaking-induced conversion (RT-QuIC) [35–37]. Since neither of these techniques have been employed on ovaries from scrapie-infected ewes, their importance in disease transmission may have been underestimated.

Therefore, the main objective of this study was to investigate the presence of prions in the oocytes and ovaries of sheep naturally infected with classical scrapie using the ultrasensitive technique PMCA. Moreover, given that multiple studies indicate that genotype [38–42] and prion strain [43–46] can affect prion accumulation in peripheral tissues, including the reproductive system, this study also sought to assess potential differences in prion accumulation in the ovaries and oocytes of sheep of two genotypes (ARQ/ARQ and VRQ/VRQ) and sheep originating from two distinct scrapie outbreaks.

## Materials and methods

### Ethics statement

This study was approved by the Ethical Advisory Commission for animal experimentation of the University of Zaragoza (identification code: P138/15) and was performed under their supervision. All procedures involving animals adhered to the guidelines included in the Spanish law for Animal Protection RD53/2013 and the European Union Directive 2010/63 on the protection of animals used for experimental purposes.

### Sample origin

Ovary samples from 25 naturally scrapie-infected ewes and 5 negative controls were analysed (Table 1) and divided into two studies. In the first study, we analysed the influence of genotype on prion accumulation in the oocytes and ovaries of 3 VRQ/VRQ and 8 ARQ/ARQ ewes from flocks in six different villages in Aragón (Spain). It was not possible to obtain and test more naturally infected VRQ/VRQ sheep due to the policies of prevention and eradication of classical scrapie (based on genetic selection of resistant breeders to reduce the prevalence of more susceptible genotypes, especially VRQ/VRQ). In the second study, the influence of the prion strain was evaluated in the oocytes and ovaries of 20 ARQ/ARQ ewes, 9 from a flock from village A and 11 from a flock from village B. Euthanasia was performed by intravenous overdose of pentobarbital. The ovaries were stored in the tissue bank of the Centro de Encefalopatías y Enfermedades Transmisibles Emergentes (University of Zaragoza, Spain), one of which was preserved frozen at −80 °C and the other in 10% formalin. In addition, as a negative control, ovaries from healthy ewes were selected from farms where no cases of classical scrapie had ever been reported.

**Table 1:** **Negative controls and naturally scrapie-affected ewes under study**

Sheep ID	Genotype	Village	Stage of disease	Age
2121	ARQ/ARQ	A	Terminal	7
2177	ARQ/ARQ	A	Terminal	4
2123	ARQ/ARQ	A	Advanced clinical	4
2124	ARQ/ARQ	A	Clinical	4
2126	ARQ/ARQ	A	Clinical	4
2158	ARQ/ARQ	A	Clinical	4
2176	ARQ/ARQ	A	Clinical	3
2125	ARQ/ARQ	A	Clinical	3
2178	ARQ/ARQ	A	Advanced clinical	3
1608	ARQ/ARQ	B	Advanced clinical	6
1611	ARQ/ARQ	B	Advanced clinical	5
1679	ARQ/ARQ	B	Advanced clinical	5
1603	ARQ/ARQ	B	Advanced clinical	4
1635	ARQ/ARQ	B	Clinical	3
1555	ARQ/ARQ	B	Clinical	2
1681	ARQ/ARQ	B	Advanced clinical	2
1685	ARQ/ARQ	B	Advanced clinical	2
1667	ARQ/ARQ	B	Advanced clinical	2
1538	ARQ/ARQ	B	Advanced clinical	1
1644	ARQ/ARQ	B	Advanced clinical	1
1670	ARQ/ARQ	C	Advanced clinical	6
1637	ARQ/ARQ	D	Clinical	3
1510	VRQ/VRQ	D	Advanced clinical	5
2370	VRQ/VRQ	E	Advanced clinical	1
1404	VRQ/VRQ	F	Clinical	4
Negative controls				
NOv1	ARQ/ARQ	G	–	3
NOv2	ARQ/ARQ	G	–	3
NOv3	ARQ/ARQ	G	–	4
NOv4	ARQ/ARQ	H	–	3
NOv5	ARQ/ARQ	H	–	3

### Oocyte retrieval and trypsinization

After the ovaries were thawed at 4 °C for 12–24 h, a Petri dish was prepared with 5 mL of 1× PBS at 37 °C for each ovary. In this dish, the ovary was dissected with sterile forceps and a scalpel. The visible follicles were punctured with a sterile needle, and longitudinal cuts were made on the external surface to extract as many oocytes as possible. A stereomicroscope was used to identify the oocytes, which were aspirated with a micropipette and deposited in PCR tubes. The oocyte plasma membrane and zona pellucida were removed by trypsinization for subsequent analysis by PMCA. For this purpose, oocytes were incubated at room temperature for 60–90 s with 5 μL of 0.25% porcine trypsin (SIGMA Trypsin–EDTA Solution (10X)) in Hanks’ balanced salt (1x), w/o Ca & Mg, w/o phenol red (400 mg/L KCl, 1000 mg/L d-glucose, 60 mg/L KH_2_PO_4_, 8000 mg/L NaCl, 350 mg/L NaHCO_3_, 48 mg/L Na_2_HPO_4_), and trypsinization was stopped by the addition of 5 μL of protease inhibitor (Complete, Sigma Aldrich).

### Prion detection in oocytes and ovarian tissue homogenates by PMCA

Prior to PMCA, ovarian tissue homogenates were prepared at 10% w/v (weight/volume) in distilled water (H_2_Od) after oocyte retrieval, ensuring that no oocytes remained in the ovary. The absence of oocytes in the remaining tissue was verified by deep cutting and manual crushing of the ovaries, followed by examination of the liquid and the released tissues with a stereomicroscope prior to their introduction into the tissue homogenizer.

Seeds (trypsinized oocytes and 10% w/v ovarian tissue homogenates) were subjected to three rounds of PMCA (24 h each), as previously reported [47]. In each well of a 96-well PCR microplate (Axygen Scientific, USA), 10 μL of seeds were mixed with 60 μL of substrate (brain homogenates from negative tg338 mice, which express the VRQ allele of ovine PrP^C^) [48]. A Teflon bead (3 mm diameter) was added to each well. Amplification was conducted using a Qsonica Q700 sonicator with a water recirculation system. The microplates were subjected to 96 PMCA cycles, each consisting of 10 s of sonication at 75% power followed by 14 min and 50 s of incubation at 42 °C. After each 24-h PMCA round, 10 μL of the reaction mixture was transferred to a new microplate containing 60 μL of fresh substrate for the next round. Serial dilutions (10^–1^ to 10^–8^) of a tg338-passaged classical scrapie isolate (Daw) [48] and a classical ovine scrapie isolate obtained from the Aragón region, were used as positive controls, whereas uninfected sheep oocytes and ovaries and unseeded substrate were used as negative controls.

PrP^Sc^ detection after PMCA was performed by dot blot. In each well of a 96-well PCR microplate, the products of the third round of PMCA were subjected to digestion and denaturation by mixing 18 μL of each product with 2 μL of 3% SDS and 5 μL of a 0.5 μg/μL dilution of Proteinase K (Roche) in the RIPA Lysis Buffer System (Santa Cruz Biotechnology). This mixture was incubated at 37 °C for 1 h, and then digestion was stopped by adding 25 μL of Laemmli buffer to each well and heating at 95 °C for 5 min. A 10-μL volume of each well was mixed with 22.5 μL of 1% SDS and 22.5 μL of 1X PBS, and the samples were then vacuum transferred onto a nitrocellulose membrane. After the membrane was blocked for 30 min with 5% milk powder in wash buffer (10 mM sodium phosphate; 0.15 M NaCl; 0.05% Tween-20; 950 mL H_2_O; pH 7.5), PrP^Sc^ immunodetection was performed with the monoclonal antibody Sha31 (mAb, 1:8000, SPI-Bio), an HRP-conjugated anti-mouse secondary antibody (1:5000; Bio-Rad) and an enhanced chemiluminescence (ECL) substrate (Pierce) to reveal peroxidase activity.

Upon completion of the dot blot procedure, western blotting was conducted to confirm positive results. Twenty microlitres of PK-digested PMCA products from the dot blot were subjected to 12% SDS‒PAGE and transferred to a PVDF membrane. Then, the membrane was blocked for 30 min with 5% milk powder in wash buffer, and the same procedure used for the dot blot was used for immunodetection of PrP^Sc^.

To quantify the signal intensity in the dot blots and western blots obtained after PMCA and assign results as “positive” or “negative”, a densiometric analysis was performed using ImageJ software (NIH). For background correction, the mean intensity of the negative controls was measured and subtracted from the test sample values. A positivity threshold was set as the mean plus two standard deviations (Mean + 2SD) of the negative controls. Samples with intensity values above this threshold were considered positive.

### Prion detection in ovaries

Tissues fixed in formalin were processed according to standard histopathological protocols. The sections (4 µm thick) were stained with haematoxylin and eosin for histopathological analysis.

The possible presence of PrP^Sc^ in ovarian tissues was evaluated via immunohistochemistry as previously described [49] using the monoclonal primary antibody L42 (1:500, R-Biopharm), which is located in the central region of the C-terminal globular domain of PrP (144FGNDYEDRYYRENMYRYPNQVYY166), in addition to the 6H4 (Prionics, Zurich, Switzerland), which recognizes a region of the C-terminus of the protein antibody (144DYDRYYRE152). Formic acid, proteinase K digestion (4 µg/mL) and hydrated autoclaving at 96 °C in citrate buffer were used for antigen retrieval.

## Results

### Prion detection in oocytes and ovarian tissue homogenates by PMCA

#### ARQ/ARQ sheep accumulate more prions in oocytes than VRQ/VRQ sheep do

**Figure 1:**
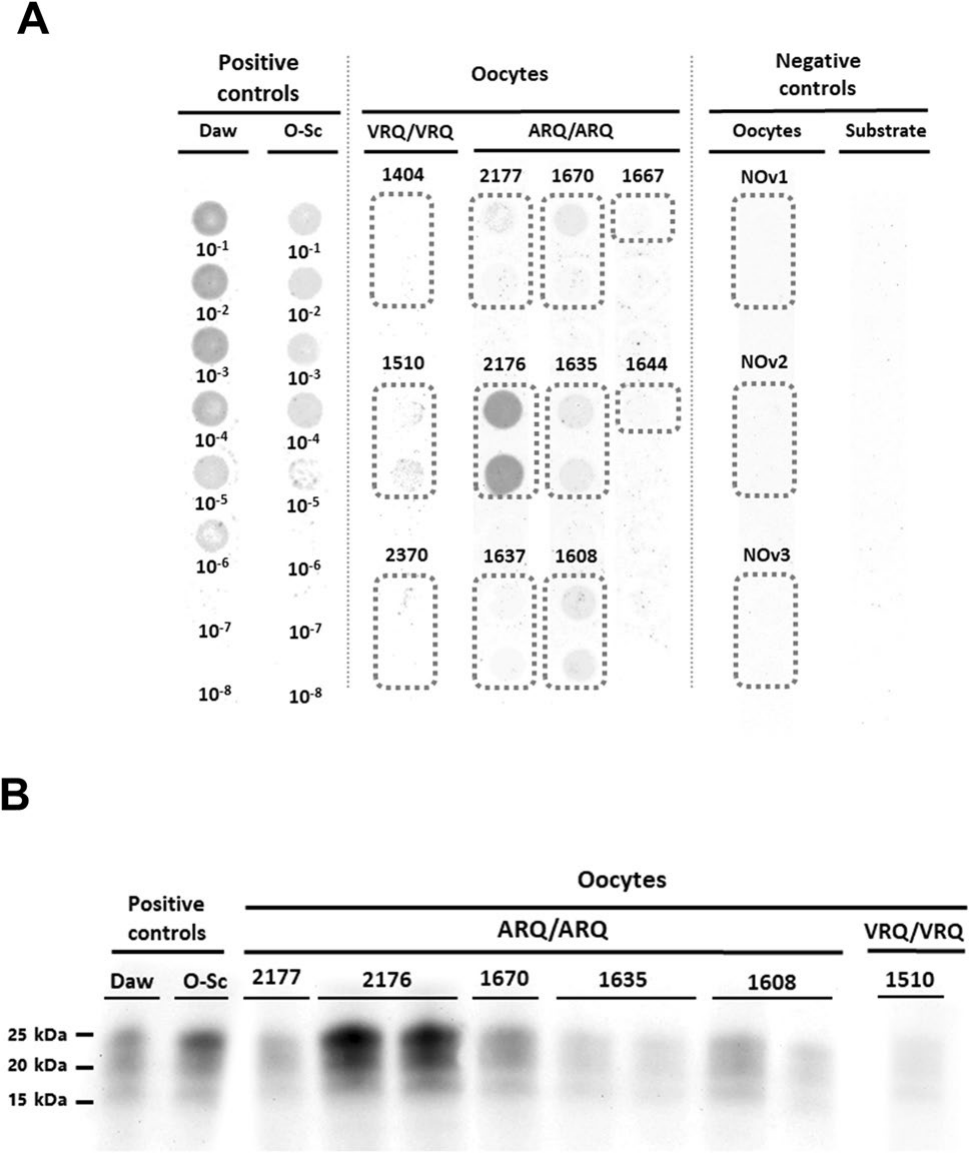
**Detection of prions present in VRQ/VRQ- and ARQ/ARQ-infected sheep oocytes.** **A** Dot blot results. Serial dilutions (10^‒1^–10^‒8^) of a tg338-passaged classical scrapie isolate (Daw) and a classical ovine scrapie isolate obtained from the Aragón region (O-Sc) were used as positive controls. Uninfected sheep oocytes (NOv1, NOv2 and NOv3) and PMCA substrate (negative tg338 mouse brain homogenate) were used as negative controls. Each ewe was analysed in duplicate, except for ewes 1667 and 1644, since it was not possible to extract more oocytes. **B** Western blot results. The results from two positive controls are included: a tg338-passaged classical scrapie isolate (Daw) and a classical ovine scrapie isolate obtained from the Aragón region (O-Sc).

In total, oocytes from 8 ARQ/ARQ and 3 VRQ/VRQ ewes were analysed. All ewes had shown clinical signs of scrapie, and most of them were in an advanced or even terminal stage of the disease (Table 1). Prion propagation after 3 rounds of PMCA was detected in the oocytes of 5/8 ARQ/ARQ ewes and only 1/3 VRQ/VRQ ewes (Figure 1A). The intensity of the dot blot signal of each propagated sample was compared with that produced by positive controls (serial 10^–1^ to 10^–8^ dilutions of a tg338-passaged classical scrapie isolate and a classical ovine scrapie isolate obtained from the Aragón region) after PMCA to estimate the amount of prions present in the oocyte samples. Oocytes obtained from healthy sheep were used as negative controls for the technique. Most oocyte samples (with the exception of 1 ARQ/ARQ sheep) were mildly positive. Seeding activity was not detected in the oocytes of healthy ewes. These results were subsequently confirmed by western blotting (Figure 1B).

#### Differences in prion accumulation and propagation in the oocytes and ovaries of ewes from different scrapie outbreaks

**Figure 2:**
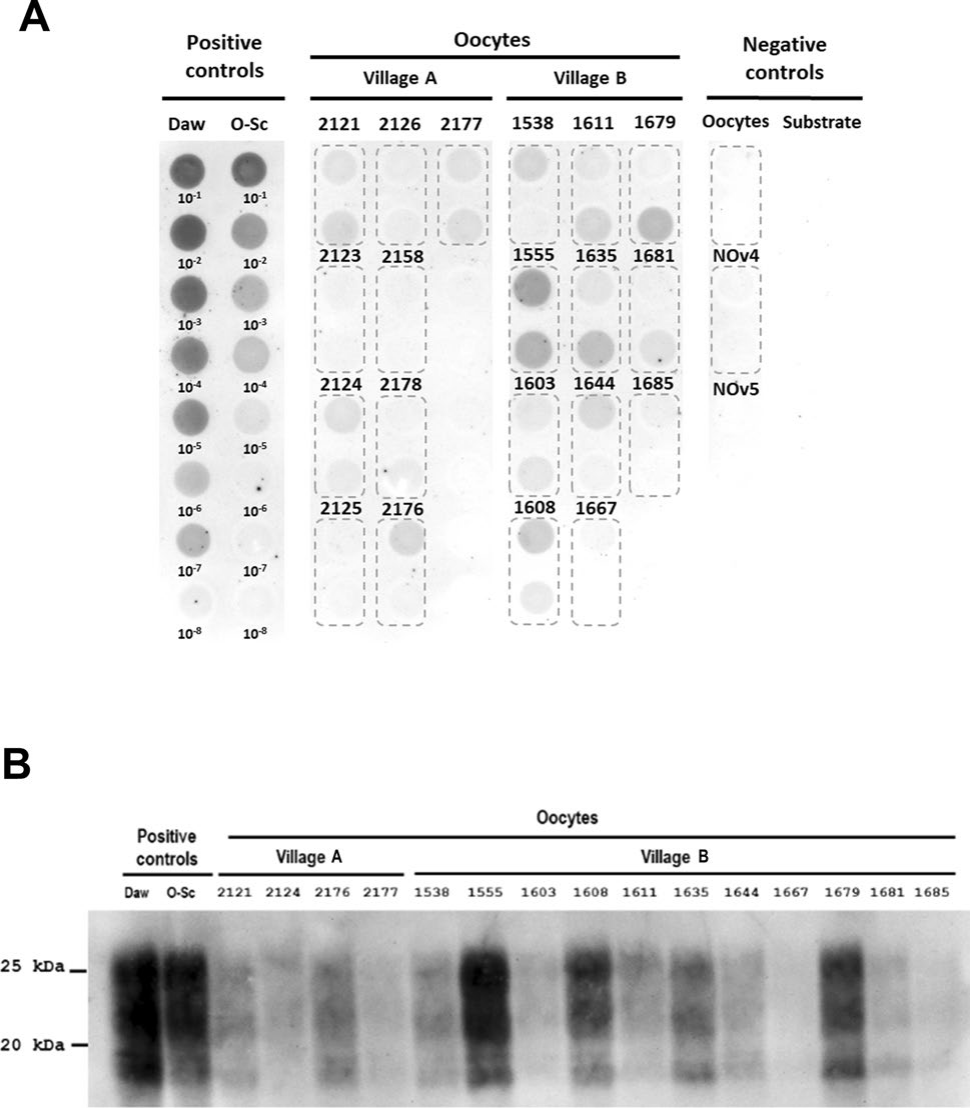
**Detection of prions present in oocytes of ARQ/ARQ-infected ewes from different scrapie outbreaks.** **A** Dot blot results. Ewes belong to flocks from two different villages in Aragón (Spain). Serial dilutions (10^‒1^–10^‒8^) of a tg338-passaged classical scrapie isolate (Daw) and a classical ovine scrapie isolate obtained from the Aragón region (O-Sc) were used as positive controls. Uninfected sheep oocytes (NOv4 and NOv5) and PMCA substrate (negative tg338 mouse brain homogenate) were used as negative controls. Each ewe was analysed in duplicate. **B** Western blot results. The results from two positive controls are included: a tg338-passaged classical scrapie isolate (Daw) and a classical ovine scrapie isolate obtained from the Aragón region (O-Sc).

**Figure 3:**
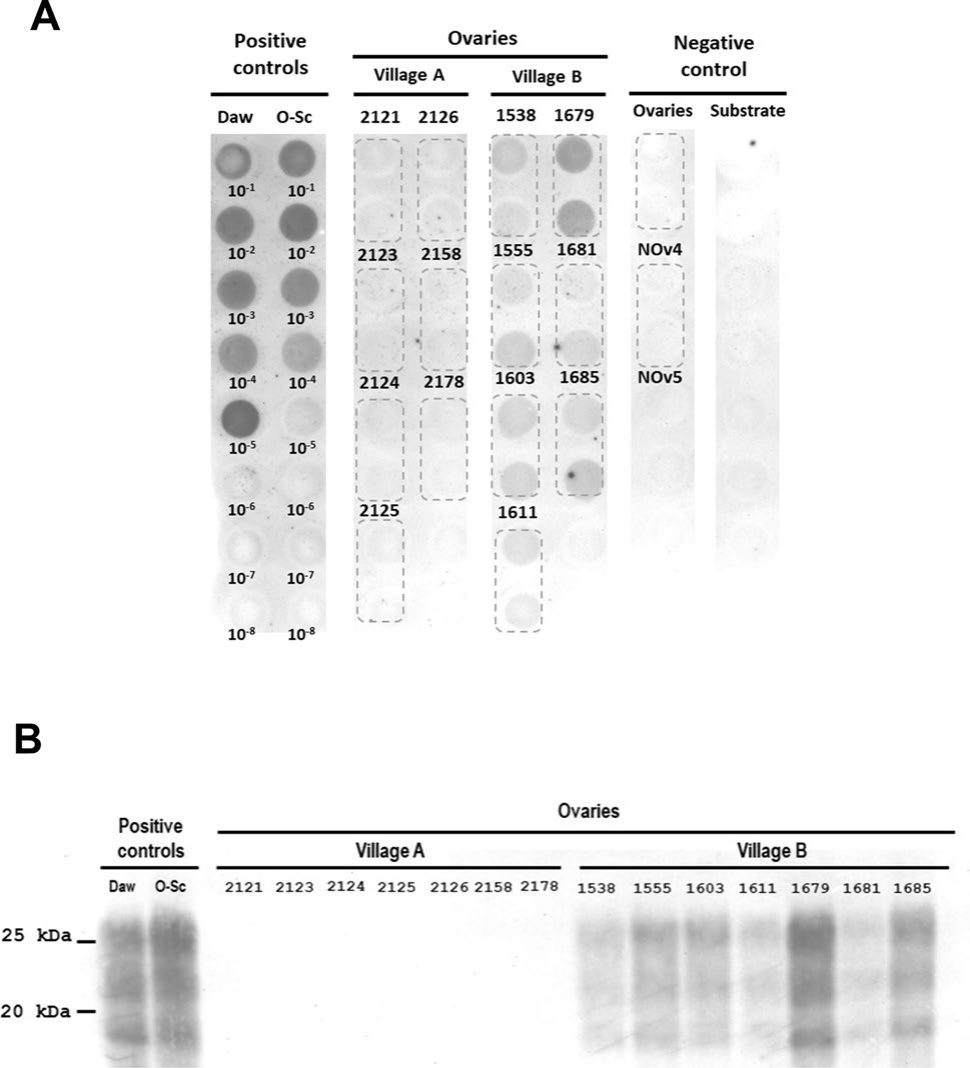
**Detection of prions present in the ovaries of ARQ/ARQ-infected ewes from different scrapie outbreaks.** **A** Dot blot results. Ewes belong to flocks from two different villages in Aragón (Spain). Serial dilutions (10^‒1^–10^‒8^) of a tg338-passaged classical scrapie isolate (Daw) and a classical ovine scrapie isolate obtained from the Aragón region (O-Sc) were used as positive controls. Uninfected sheep ovaries (NOv4 and NOv5) and PMCA substrate (negative tg338 mouse brain homogenate) were used as negative controls. Each ewe was analysed in duplicate. **B** Western blot results. The results from two positive controls are included: a tg338-passaged classical scrapie isolate (Daw) and a classical ovine scrapie isolate obtained from the Aragón region (O-Sc).

To test the role of the prion isolate in oocyte prion accumulation, seeding activity in oocytes from 20 naturally infected ARQ/ARQ ewes in flocks from two different villages was analysed by PMCA. Nine sheep were obtained from Village A, and 11 were obtained from Village B. The PMCA results were analysed by dot blotting, following a semiquantitative approach, and the seeding activity of each sample was compared with that of positive controls (serial 10^–1^ to 10^–8^ dilutions of a tg338-passaged classical scrapie isolate and a classical ovine scrapie isolate obtained from the Aragón region) and negative controls (uninfected PMCA substrate and oocytes from healthy ewes) (Figure 2A). Ovaries from 14 of these ewes were also analysed following the same procedure; however, in this case, ovaries from healthy ewes were used as negative controls (Figure 3A). All the ewes tested presented with clinical signs of scrapie, and most of them were in an advanced or even terminal stage of the disease (Table 1).

Oocyte analysis revealed 5/9 positive results in sheep from Village A and 11/11 positive results in sheep from Village B. In addition, the relative amount of PrP^Sc^ obtained in the oocytes of Village B after PMCA was greater than that of Village A (Figure 2A). Moreover, regarding ovarian tissue, no positive result was observed in Village A, as opposed to Village B, where all the ovaries analysed were positive for prion propagation (Figure 3A). PrP^Sc^ was not detected in the oocytes or ovaries of healthy ewes. These results were subsequently confirmed by western blotting (Figure 2B and Figure 3B).

No PrP^Sc^ could be detected by immunohistochemistry in the ovarian tissues from any of the VRQ/VRQ or ARQ/ARQ ewes included in the present study (Additional file 1).

## Discussion

To our knowledge, this is the first report describing the presence of PrP^Sc^ in oocytes in naturally acquired classical scrapie. Several studies, however, have previously detected the presence of *PRNP* and PrP mRNA in various reproductive tissues and related cells, including ovaries, ovarian follicles, theca and granulosa cells and oocytes (especially immature ones) [19, 27, 50, 51], indicating that PrP^Sc^ has the necessary substrate to replicate in oocytes. In fact, it has been suggested that PrP^C^ may play an important role in promoting ovarian follicle development [50]. Consequently, prion accumulation in oocytes seems plausible, which could indicate transmission through the germline.

On the other hand, certain differences in prion accumulation according to genotype were observed, as prion propagation seems to be detected in a larger number of oocyte samples from ARQ/ARQ ewes (Figure 1), although the VRQ/VRQ genotype is more susceptible to pathology. In addition, prion amplification in ARQ/ARQ oocytes was relatively more intense. It is widely known that cell specificity, kinetics and tissue distribution are influenced by different factors, with the *PRNP* genotype being one of the most significant [38, 39]. The *PRNP* genotype also determines susceptibility to infection. Previous studies have shown that VRQ/VRQ ewes have not only shorter incubation periods but also greater tissue dissemination of PrP^Sc^ than do ARQ/ARQ ewes in nervous and lymphoid tissues [52–55]. However, this superior peripheral dissemination of PrP^Sc^ in VRQ/VRQ sheep might not occur in other tissues. For example, in a previous study on the presence of prions in foetuses of naturally infected sheep, the VRQ/VRQ genotype was associated with a very low level of infectivity and negative results in almost all the foetuses tested [16], whereas in another study, all the ARQ/ARQ foetuses tested were positive for prion accumulation [15]. Similarly, other authors reported extensive dissemination of PrP^Sc^ in peripheral organs outside the CNS and LRS of naturally infected ARQ/ARQ ewes, whereas VRQ/VRQ ewes were negative. These results were attributed to the slower progression of the pathology in ARQ/ARQ sheep than in VRQ/VRQ sheep, particularly in older animals. The extended incubation period in ARQ/ARQ sheep allows more time for PrP^Sc^ to disseminate throughout various peripheral organs [26].

However, in these studies, only a small number of VRQ sheep were analysed, and the same applies to the present study, in which sheep were also obtained from different geographical areas. Therefore, although differences were observed between ARQ/ARQ and VRQ/VRQ sheep, it is not possible to draw definitive conclusions about the effect of genotype on PrP^Sc^ accumulation in oocytes, as these disparities may also be linked to variations in prion strains affecting these flocks, which can affect peripheral PrP^Sc^ accumulation [43–46]. Therefore, to investigate the influence of prion strains/isolates on prion accumulation in oocytes, seeding activity was analysed in oocytes collected from 20 ARQ/ARQ ewes from two distinct flocks located in 2 villages separated by 131 kms. Greater accumulation of PrP^Sc^ was detected in the oocytes and ovaries of ewes from Village B than in those from Village A. To our knowledge, this is the first report describing the presence of PrP^Sc^ in ovaries in naturally acquired classical scrapie by PMCA.

The ewes from both villages had the ARQ/ARQ genotype and were at approximately the same stage of pathology. However, the age of the affected sheep differed between flocks. Six of the eight ewes from Village A were ≥ 4 years old, whereas the scrapie-affected sheep from Village B were significantly younger (Table 1). Since we studied naturally infected sheep, we cannot determine the exact moment at which the animals were infected or, therefore, the incubation period. However, the young age of clinical sheep from Village B, together with the differential prion accumulation in oocytes and ovaries observed, suggests that these geographically distant outbreaks might be caused by different scrapie strains. In addition, the scrapie strain present in Village B may be more aggressive or better adapted to sheep of the ARQ/ARQ genotype. However, characterization studies in different mouse lines are needed to determine whether these outbreaks are caused by distinct strains. On the other hand, the results obtained in sheep from Village A suggest that prions either accumulate in oocytes or ovarian follicles at a relatively high rate or that this accumulation occurs earlier than their spread to the rest of the ovarian tissue, as oocytes tested positive when all ovarian samples were negative (Table 2). The exact reason for this remains unknown. This could be due to different levels of PrP^C^ expression in the ovary and ovarian follicles, given the suggested importance of PrP^C^ in follicular development [50] and the fact that prion tropism is also influenced by the differential expression of PrP^C^ in different organs [56]. Similar results have been reported in other studies. For example, a study on CWD vertical transmission in deer suggested that prions accumulate at the maternal‒foetal interface rather than in maternal reproductive organs, as PrP^Sc^ was detected in the placentomes of infected females whose uterus and ovaries were negative by RT-QuIC [37]. Similarly, in another study, 6-day-old embryos from scrapie-infected ewes were transferred and implanted in healthy ewes, resulting in the development of the disease in offspring, which suggests that transmission occurs in the early stages of embryogenesis or with the germline [17].

**Table 2:** **Detection of PrP**^**Sc**^ **by PMCA and immunohistochemistry (IHC) in oocytes and ovaries of ARQ/ARQ and VRQ/VRQ ewes**

Sheep ID	Oocytes	Ovaries	
	PMCA	PMCA	IHC
2121	+	−	−
2177	+	−	−
2123	−	−	−
2124	+	−	−
2126	−	−	−
2158	−	−	−
2176	+	−	−
2125	−	−	−
2178	+	−	−
1608	+	Not tested	−
1611	+	+	−
1679	+	+	−
1603	+	+	−
1635	+	Not tested	−
1555	+	+	−
1681	+	+	−
1685	+	+	−
1667	+	Not tested	−
1538	+	+	−
1644	+	Not tested	−
1670	+	Not tested	−
1637	−	Not tested	−
1510	+	Not tested	−
2370	−	Not tested	−
1404	−	Not tested	−
**Negative controls**			
NOv1	−	Not tested	−
NOv2	−	Not tested	−
NOv3	−	Not tested	−
NOv4	−	−	−
NOv5	−	−	−

Additionally, no PrP^Sc^ deposits were detected by immunohistochemistry in any of the ovaries, even those that tested positive for PMCA (Additional file 1). These results coincide with those of previous studies, where it was not possible to detect PrP^Sc^ by immunohistochemistry or ELISA in the ovaries of ewes with scrapie [26] or in the ovaries of deer with CWD. However, in the case of CWD-infected does, this was possible by PMCA, RT-QuIC and bioassays [37]. Consequently, it is deduced that prion accumulation in ovaries is too low to be detected by conventional techniques, and it is necessary to resort to ultrasensitive techniques for this purpose. This fact is logical considering that the neurons innervating the reproductive organs are associated with the most caudal segment of the spinal cord, the sacrum, which is located farther from the initial sites of invasion. Consequently, since prion infection progresses slowly from the thoracic part of the CNS to the sacral region, reproductive organs are among the last organs to be infected [26].

Our results show that PrP^Sc^ accumulation in the oocytes and ovaries of ewes naturally infected with classical scrapie is possible, suggesting potential transmission of the disease through the germ line. However, prion accumulation is too low to be detected by conventional techniques, and ultrasensitive techniques such as PMCA are necessary. These results highlight the importance of these techniques for future studies on the involvement of reproductive organs in the vertical transmission of scrapie. In addition, differences in prion accumulation in oocytes and ovaries may be associated with the prion strain involved and the genotype of the host. However, further studies are needed to analyse these factors under experimental conditions, with characterized prion strains and known incubation periods. Consequently, the findings of the present study highlight the need to continue investigating the possible routes of scrapie vertical transmission, as it may be necessary to implement new control and selection strategies in breeding programs to reduce the spread of the disease.

## Supplementary Information

**Additional file 1. Immunohistochemical (IHC) detection of PrP**^**Sc**^** in ovaries (A) and haematoxylin‒eosin (HE) staining (B).** No PrP^Sc^ deposits were detected in any of the ovaries analysed, not even those positive for PMCA. For example, the IHC (mAb L42; ×50) (**A**) and HE (×50) (**B**) results of VRQ/VRQ 1510 ewe ovaries (positive for classical scrapie) are included. No differences were found in the IHC and HE results from the ovaries of infected ARQ/ARQ ewes or negative controls.

## Data Availability

All the data generated or analysed during this study are included in this published article.
