# Supplementary figures and images for: Detection of prions in oocytes and ovaries of ewes naturally infected with classical scrapie
Source: Vet Res. 2025 Apr 10;56:79. doi: 10.1186/s13567-025-01512-0 (PMC11984232; doi:10.1186/s13567-025-01512-0)

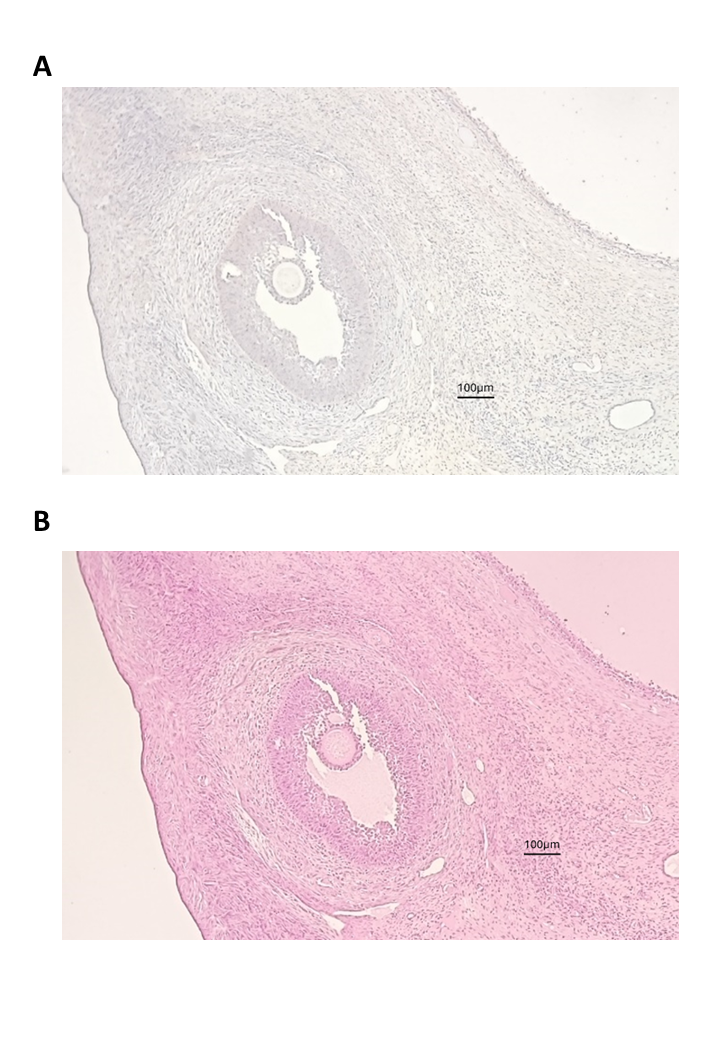

Supplement: Supplementary file 1 — Additional file 1. Immunohistochemical (IHC) detection of PrPSc in ovaries (A) and haematoxylin‒eosin (HE) staining (B). No PrPSc deposits were detected in any of the ovaries analysed, not even those positive for PMCA. For example, the IHC (mAb L42; ×50) (A) and HE (×50) (B) results of VRQ/VRQ 1510 ewe ovaries (positive for classical scrapie) are included. No differences were found in the IHC and HE results from the ovaries of infected ARQ/ARQ ewes or negative controls. [file 13567_2025_1512_MOESM1_ESM.tiff]
